# A Monocular Camera as an Operation Logger for Motorized Mobility Scooters: Visual Odometry Method to Estimate Steering and Throttle Angles

**DOI:** 10.3390/s25092701

**Published:** 2025-04-24

**Authors:** Naoto Haraguchi, Yi Liu, Haruki Sugiyama, Kazunori Hase, Jun Suzurikawa

**Affiliations:** 1 Department of Assistive Technology, Research Institute of National Rehabilitation Center for Persons with Disabilities, 4-1 Namiki, Tokorozawa 359-8555, Japan; haraguchi-naoto@rehab.go.jp; 2 College of Mechanical and Electrical Engineering, Harbin Engineering University, 145 Nantong Street, Nangang District, Harbin 150001, China; liu-yi.mech@hrbeu.edu.cn; 3 Department of Mechanical Systems Engineering, Tokyo Metropolitan University, 6-6 Asahigaoka, Hino 191-0065, Japan; sugiyamaharuki98@gmail.com (H.S.); kazunori.hase@tmu.ac.jp (K.H.)

**Keywords:** image processing, optical flow, template matching, Lucas-Kanade method, assistive technology

## Abstract

Motorized mobility scooters (MMSs) are vital assistive technology devices that facilitate independent living for older adults. In many cases, older adults with physical impairments operate MMSs without special licenses, increasing the risk of accidents caused by operational errors. Although sensing systems have been developed to record MMS operations and evaluate driving skills, they face challenges in clinical applications because of the complexity of installing inertial measurement units (IMUs). This study proposes a novel recording system for MMS operation that uses a compact single-lens camera and image processing. The system estimates steering and throttle angles during MMS operation using optical flow and template matching approaches. Estimation relies on road surface images captured by a single monocular camera, significantly reducing the complexity of the sensor setup. The proposed system successfully estimated the steering angle with comparable accuracy to existing approaches using IMUs. Estimation of the throttle angle was negatively affected by the inertia of the MMS body during acceleration and deceleration but demonstrated high accuracy during stable driving conditions. This method provides a fundamental computational technique for measuring MMS operations using camera images. With its simple setup, the proposed system enhances the usability of recording systems for evaluating MMS driving skills.

## 1. Introduction

Dysfunction of the lower limbs can significantly impair ambulation in older adults and those with physical disabilities. Impaired mobility reduces the level of activities of daily living and limits social participation, ultimately lowering quality of life [1,2]. Assistive technology devices (ATDs), including canes, walkers, and wheelchairs, have been introduced as mobility aids for use inside and outside the home to compensate for reduced physical function. These ATDs enable autonomous mobility and foster active social participation [3,4,5].

Motorized mobility scooters (MMS) are among the most commonly used ATDs for enhancing mobility, particularly among older adults with frailty or motor impairments [6,7,8,9]. Compared with power wheelchairs (PWCs), MMSs offer advantages in stable driving in outdoor environments. Unlike PWCs, MMSs are not equipped with casters, allowing for easier maintenance of a straight trajectory on various terrains. Moreover, the steering tiller, positioned in the front of the driver’s seat, helps users maintain an appropriate posture while driving. These features contribute to the active outdoor use of MMSs among older adults [10].

However, the increased use of MMSs raises safety concerns in local communities with automobile traffic and environmental obstacles [6,11]. In most countries and regions, MMSs can be operated without special licenses or approvals [12,13]. The primary users of MMSs often experience declining cognitive and motor functions, highlighting the need for continuous assessment of their driving skills, monitoring usage conditions, and guidance to ensure safe operation. Although such interventions are commonly implemented for manual and power wheelchairs [14], comprehensive intervention processes and methods for MMSs remain underdeveloped.

Recent advancements in sensing technologies have introduced innovative techniques for monitoring and assessing activities with PWCs and MMSs. Installing inertial measurement units (IMUs) on wheelchair bodies, combined with machine-learning-based classification of the collected data, enables quantifying daily activities in real-life environments [15]. Recording and analyzing joystick operation inputs are valuable for assessing driving skills and stability [16]. Although most commercially available PWCs do not feature built-in operation logging capabilities, add-on IMU-based logging systems have been developed to characterize driving tendencies and maneuvers. In our previous study, we applied IMU-based operation logging to MMSs and proposed a method for estimating the operation angles of the throttle lever and steering wheel [17]. Such easy-to-install monitoring systems can contribute to the quantitative assessment of driving activities and promote the safe use of MMSs.

Despite their potential, IMU-based activity monitoring systems for MMSs face practical implementation challenges [15,16,17]. The accuracy of operation estimation methods is highly dependent on sensor alignment, as they rely on gravitational acceleration as a reference. Moreover, sensor implementation including attachment fixation on the vehicle body and wiring between IMUs and data loggers can be problematic, particularly in settings where engineering staff are unavailable. Furthermore, the design of MMS operation interfaces, where IMUs are typically mounted using customized jigs, varies among manufacturers and models. The shortage of human resources in aging societies exacerbates these challenges, making it difficult to implement complex monitoring procedures even when necessary to ensure safe MMS operation.

An emerging technique for monitoring PWC/MMS activities involves the use of image processing with video cameras as sensing devices [18,19,20]. In particular, visual odometry (VO), which was originally developed for Simultaneous Localization and Mapping (SLAM) [21], was used for estimating vehicle motion from sequential video frames. Image analysis of the captured environment during driving enables the quantification of vehicle activities, including driving trajectory, motion, and stability. For example, Wolkowicz and colleagues proposed a VO-based method incorporating the extended Kalman filter for detection of PWC slip on icy or low-friction surfaces [19]. Jayasuriya and colleagues developed a vision-only SLAM system mounted on MMSs as a step toward low-cost autonomous driving technology for personal mobility devices [20]. However, previous studies have not focused on the quantification of driving operations or the evaluation of driving skills.

In this study, we propose and validate a novel method for monitoring MMS operations using VO. VO processes image sequences to estimate the vehicle’s velocity vector by analyzing video frames of the road terrain or surrounding environment. While VO has been widely used to estimate vehicle motion, the novelty of this study lies in the development of a conversion equation to obtain operation angles of the steering tiller and throttle lever from the VO-derived velocity vector. This equation was constructed based on the geometric structure of the MMS and is a key contribution of the present study. A compact single-lens video camera was used for terrain image capture, selected for its small form factor, high frame rate, and wide field of view, which enhance its suitability for recording terrain details under diverse driving conditions. The accuracy of the proposed method was validated through driving experiments conducted on various terrains. Furthermore, two major VO techniques—template matching (TM) and optical flow (OF) algorithms [22]—were compared to assess their robustness across different terrain conditions.

## 2. Materials and Methods

### 2.1. Operation Logging System

**Figure 1:**
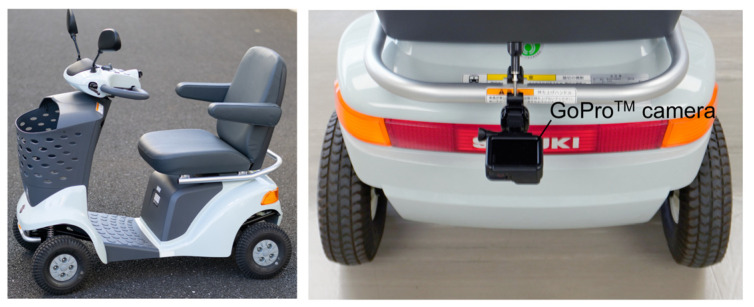
Motorized mobility scooter equipped with a monocular camera.

This study employed the MMS (ET-4D; Suzuki Motor Corp., Shizuoka, Japan), which has the top market share in Japan (Figure 1). This scooter includes a steering wheel and throttle levers in its front panel. The steering wheel is connected to the front wheels via a steering shaft with a simple mechanical linkage. The left and right throttle levers share a rotary shaft. An electric motor, connected to the rear wheels, is driven according to the rotational angle of the rotary shaft. Drivers operate the steering wheel and the throttle levers using their hands and fingers, respectively.

The GoPro^TM^ (HERO7 Black; GoPro Inc., San Mateo, CA, USA) was used to capture video of the road surface while driving the MMS. It was mounted on the rear bar of the MMS using a specialized mount (Pole Mount; GoPro Inc., San Mateo, CA, USA, Figure 1). The camera lenses were aligned with the midpoints of the left and right wheels in a horizontal position relative to the ground and the rear wheel shaft. The camera settings were as follows: resolution: 1920 × 1080 pixels, frame rate: 120 fps, field of view: linear, shutter speed: 1/480 s, ISO sensitivity: 400, white balance: 5000 K, sharpness: medium. The linear mode for the field of view in the GoPro^TM^ camera setting was chosen to eliminate distortions caused by the camera. After mounting the camera on the MMS, calibration was performed using images of a checkerboard placed on the ground, which computed the 2-dimensional (up/down and right/left directions of the image) calibration coefficients to convert image dimensions into real-world measurements. The camera calibration was conducted using the Computer Vision Toolbox^TM^ in MATLAB^®^ 24.2 (MathWorks, Inc., Natick, MA, USA).

### 2.2. Monocular Camera Image Processing

The steering and throttle angles were estimated based on the images captured by the monocular camera as follows: (i) calculate the feature point velocity in the images using VO, and (ii) estimate the steering and throttle angles from the feature point velocity based on a geometry of the MMS operation as explained in Section 2.3. Because VO techniques for vehicles include various features depending on the type of approach, this study employed two popular methods—OF approach and TM approach—to compare their estimation accuracy. The TM approach performs a cross-correlation analysis between the luminance distribution of the input image and that of the template image [23]. The cross-correlation coefficient (*CC*) between the template image and the input image at position (*x*, *y*) was calculated as follows:

(1) $$CC\left(x,y\right)=\frac{\sum_{i=0}^{n-1}\sum_{j=0}^{n-1}\left(I\left(x+i,y+j\right)-\mu_{I}\left(x,y\right)\right)\left(T\left(i,j\right)-\mu_{T}\right)}{\sqrt{\sum_{i=0}^{n-1}\sum_{j=0}^{n-1}{\left(I\left(x+i,y+j\right)-\mu_{I}\left(x,y\right)\right)}^{2}}\sqrt{\sum_{i=0}^{n-1}\sum_{j=0}^{n-1}{\left(T\left(i,j\right)-\mu_{T}\right)}^{2}}},$$

where *I* and *T* represent the luminance values of the input image and the template image, respectively, and *μ*_I_ and *μ*_T_ are the average luminance of the input and the template images, respectively. In this study, the window size was defined as *n* = 200 pixels. The TM approach determines the feature point velocity based on the shift of the pixel with the highest cross-correlation coefficient. While the TM method has the advantage of relatively simple computation requiring minimal parameter tuning, it is susceptible to the influence of noise, such as shadows in the image [24].

The OF approach is widely used as a robust VO technique because of its noise resilience and ability to calculate the corner point flow with sub-pixel accuracy [21]. This method extracts feature points from an image and computes the feature point velocity based on the luminance changes of locations at these corner points. This study employed the Shi-Tomasi corner detection algorithm to extract the feature points [25]. In addition, the OF approach uses the brightness constancy assumption for feature points located at (*x*, *y*) at time *t* [26], as follows:

(2) $$I\left(x,y,t\right)=I\left(x+∆x,y+∆y,t+∆t\right).$$

Based on Equation (2), the feature point velocity (Δ*x*/Δ*t*, Δ*y*/Δ*t*) was determined using Taylor expansion, as follows:

(3) $$\frac{\partial I}{\partial x}\frac{∆x}{∆t}+\frac{\partial I}{\partial y}\frac{∆y}{∆t}+\frac{\partial I}{\partial t}=0\;.$$

In this study, the Lucas-Kanade method was applied to solve the Equation (3) using the least squares method, based on the luminance changes of several points adjacent to the feature point [27]. All VO processes were performed using the OpenCV library version 4.10.0.

The feature point velocity was calculated as the median velocity of 27 points within the image to prevent the negative impact of noise, such as object shadows in the image. The TM approach placed 27 windows in the image and calculated the velocity of each window. In the OF approach, the Shi-Tomasi corner detection extracted 27 points from the image, and the Lucas-Kanade method computed the velocity of each point. After the velocity computation by the VO, the feature point velocity was converted in units of pixels to the actual measurement using the calibration coefficients (*k_x_*, *k_y_*) obtained by camera calibration, as follows:

(4) $$p_{x}=k_{x}x\;,\;p_{y}=k_{y}y\;,v_{x}=k_{x}\frac{∆x}{∆t}\;,\;v_{y}=k_{y}\frac{∆y}{∆t}\;.$$

### 2.3. Geometric Estimation of Driving Operations

**Figure 2:**
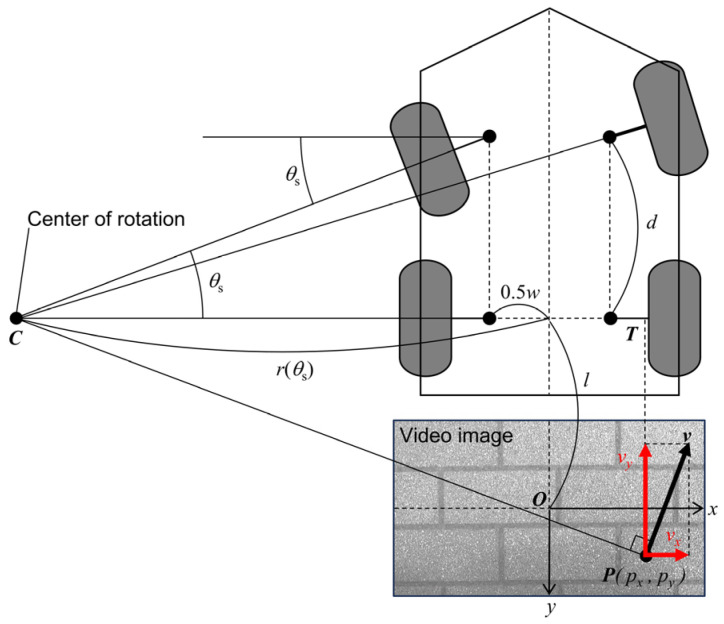
Geometry of the motorized mobility scooter during operation. The radius of curvature (*ρ*) is determined based on the similarity between the feature point velocity (*v_x_*, *v_y_*) and triangle CPT formed by the feature point position (*p_x_*, *p_y_*), the rear shaft-to-camera lens distance (*l*), and *ρ*. The steering angle (*θ*_s_) is calculated using the geometric relationship between the left-to-right wheel distance (*w*), front-to-rear wheel distance (*d*), and *ρ*.

The steering system of the MMS consisted of a simple mechanical linkage that directly connects the steering wheel to the front wheels. Therefore, the steering angle was estimated based on the MMS model with reference to the Ackermann steering geometry, depicted in Figure 2. This method was characterized by robustness against drift caused by cumulative errors, as the steering and throttle angles were calculated without relying on integral calculations. The radius of curvature (*r*) was defined as a function of the steering angle (*θ*_s_) as follows:

(5) $$r\left(\theta_{s}\right)=\frac{d}{\tan\theta_{s}}+0.5w\;,$$

where *d* is the distance between the front and rear wheels, and *w* is the distance between the left and right wheels. The radius of curvature was also determined based on the similarity between the feature point velocity (*v_x_*, *v_y_*) and the triangle formed by the feature point position (*p_x_*, *p_y_*), the center of rotation, and the rear wheel shaft, as follows:

(6) $$r(\theta_{s})=\frac{v_{y}}{v_{x}}\left(l+p_{y}\right)-p_{x}\;,$$

where *l* is the distance between the rear wheel shaft and the center of the camera lens. Based on Equations (5) and (6), the steering angle was estimated as follows:

(7) $$\theta_{s}=\tan^{-1}\left(\frac{v_{x}d}{v_{y}\left(l+p_{y}\right)-v_{x}(0.5w+p_{x})}\right)\;.$$

The throttle angle was calculated based on the assumption that the throttle angle (*θ*_t_) is proportional to the feature point velocity *v_y_*, as follows:

(8) $$\theta_{t}=\frac{v_{y}}{v_{y\_max}}\;\theta_{t\_max}\;,$$

where *v_y_*__max_ and *θ*_t_max_ are the maximum velocity and the maximum throttle angle, respectively, which were set to *v_y_*__max_ = 1.11 m/s and *θ*_t_max_ = 32 deg determined by the MMS characteristics. The steering and throttle angles were smoothed by a median filter with a 50-frame window size to remove the spike noise caused by MMS vibration.

### 2.4. Experimental Procedures

This study recruited four volunteers (mean age: 22.3 ± 1.5 years; mean height: 1.69 ± 0.08 m; mean weight: 65.3 ± 7.6 kg) with no history of musculoskeletal disorders. The study was approved by the Institutional Review Board of the National Rehabilitation Center for Persons with Disabilities. All participants received verbal and written explanations regarding the study and provided written informed consent.

**Figure 3:**
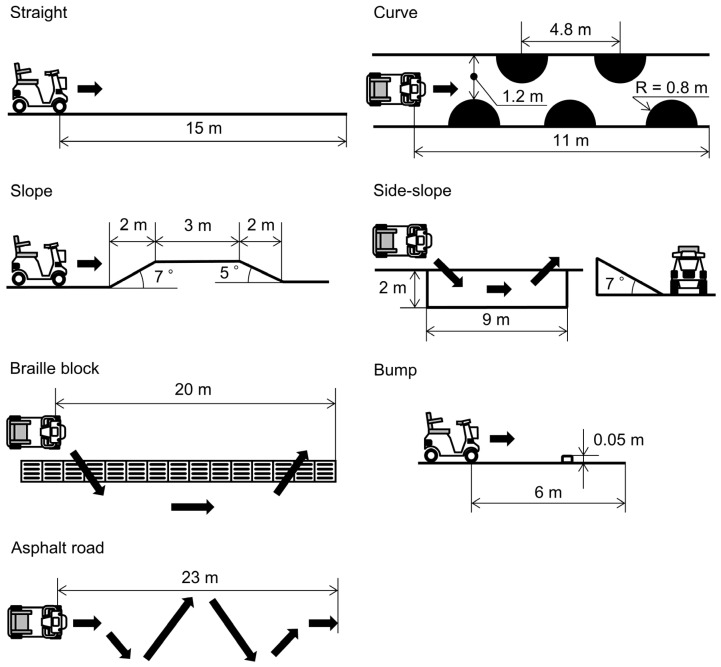
Seven courses set up for the experiment. The asphalt road surfaces consist of fine asphalt, the braille block surfaces are made of tile, and the other surfaces are rough asphalt.

The experiment was conducted on an outdoor wheelchair training field at the National Rehabilitation Center for Persons with Disabilities, designed in accordance with the wheelchair skills test [28]. Seven courses were selected to represent conditions typically encountered when operating the MMS: straight, curve, slope, side-slope, braille block, bump, and asphalt road (Figure 3). The surface materials varied among courses, including fine asphalt for the asphalt road, tiles for the braille block, and rough asphalt for the other courses. The participants traveled through the courses on the MMS set at a maximum speed of 4 km/h, the same as the average human walking speed.

**Figure 4:**
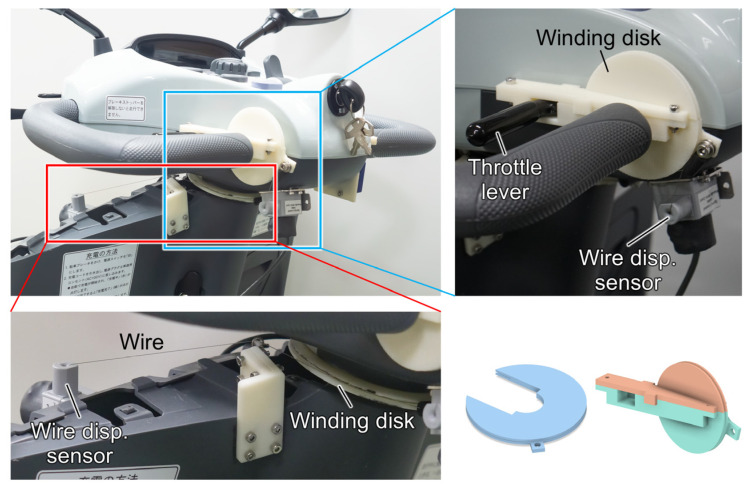
Wire displacement sensors for recording the true values of the steering and throttle angles, referring to a previous study [17].

Wire displacement sensors (SM2; TE Connectivity Ltd., Schaffhausen, Switzerland) were used to measure the operating angles of the MMS as true values to validate the estimation results [17] (Figure 4). These sensors were connected to the steering wheel and throttle levers via wire-winding disks, recording the actual operating angles based on the amount of wire wound during the steering and throttle operation. The estimated steering and throttle angle were compared with the true values to assess the accuracy of the estimation. The agreement between the estimated and true values was evaluated using the 90th percentile of the absolute error. In addition, an IMU sensor (LPMS CU2; LP-Research Inc., Tokyo, Japan) was attached to the MMS body to measure the acceleration in the vehicle’s forward direction. To investigate the effect of vehicle inertia on the estimation results, this study performed a correlation analysis between the estimation errors and the forward acceleration during MMS operation. Spearman’s rank correlation coefficient was used for the analysis due to the non-normality of the data, as determined by the Anderson–Darling test. All statistical analyses were conducted using R version 4.4.2, with *p* < 0.05 indicating statistical significance.

## 3. Results

**Figure 5:**
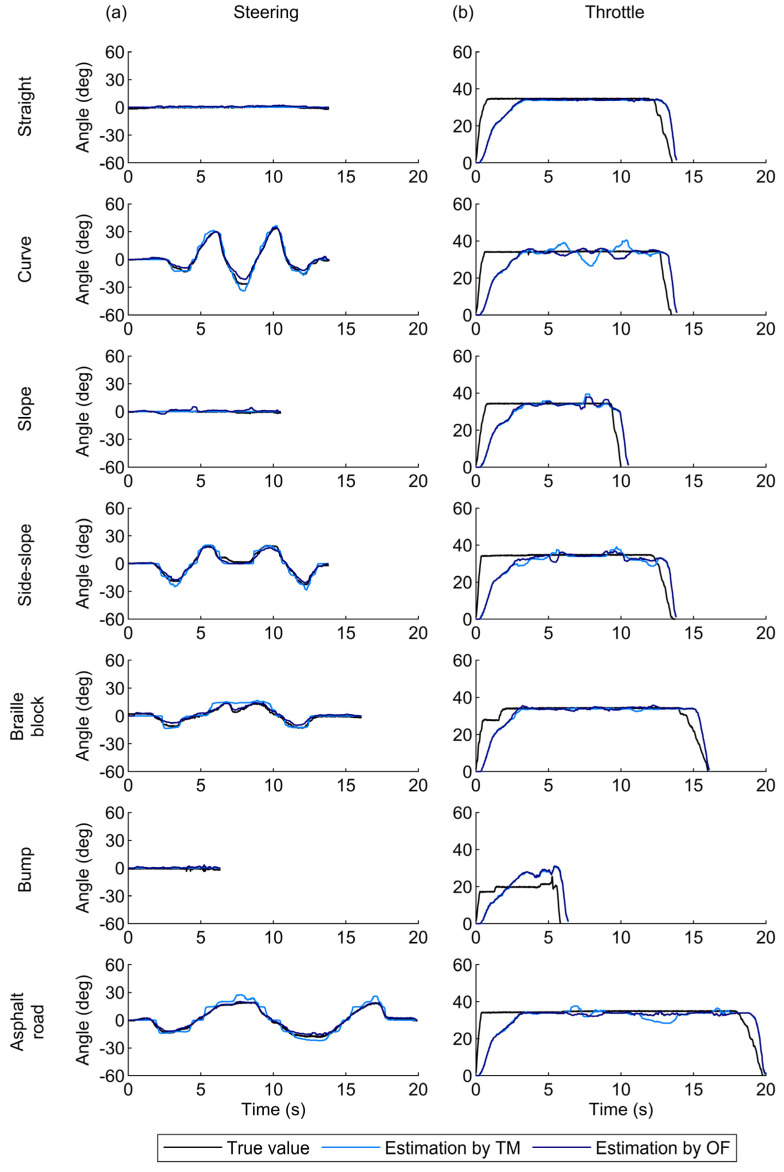
Time-series plots of (**a**) the steering angle and (**b**) the throttle angle for one participant. The dark and light blue lines represent the estimated data obtained using the optical flow (OF) and template matching (TM) approaches, respectively. The true values obtained the wire displacement sensors are represented by black lines.

**Figure 6:**
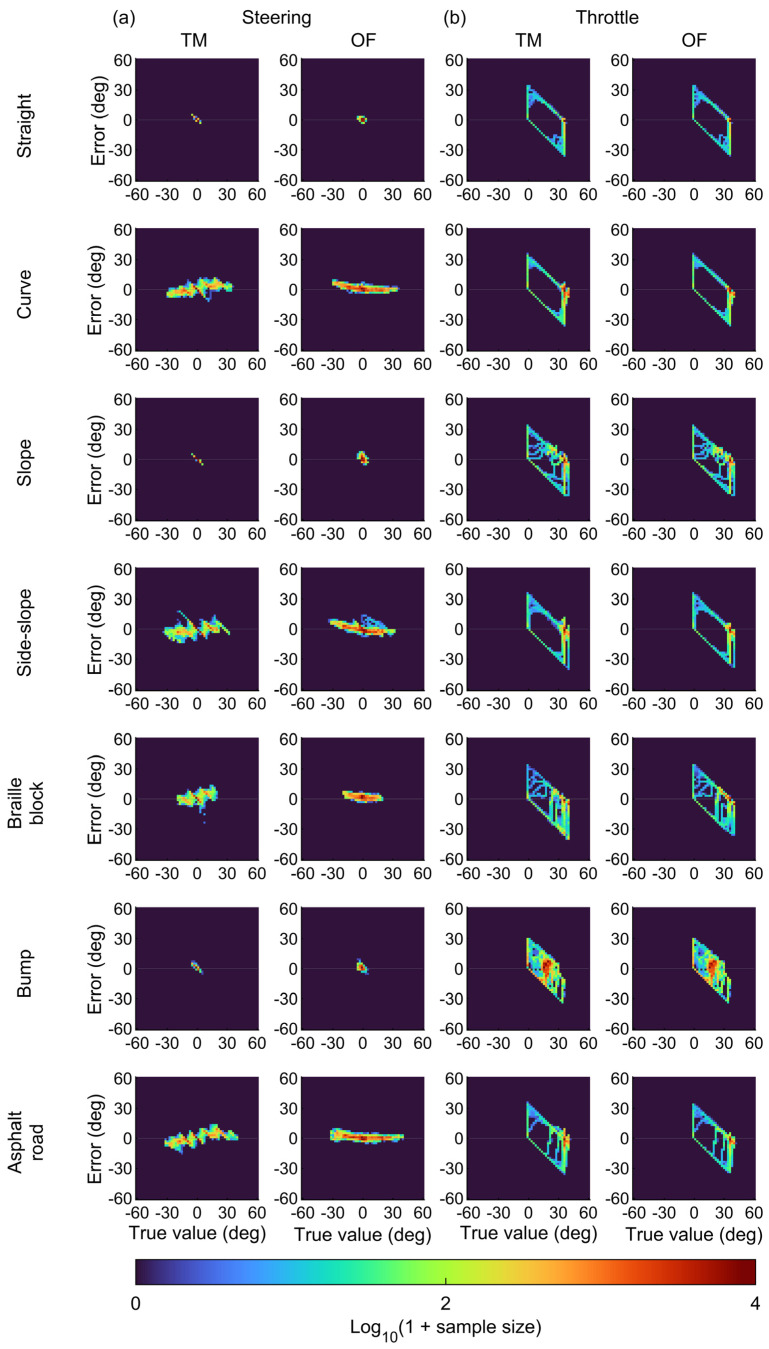
Two-dimensional histograms of the errors between the estimation and true value (i.e., estimated value minus true value) with respect to the true values for (**a**) the steering angle and (**b**) the throttle angle. The estimated values were obtained using the optical flow (OF) and template matching (TM) approaches.

**Figure 7:**
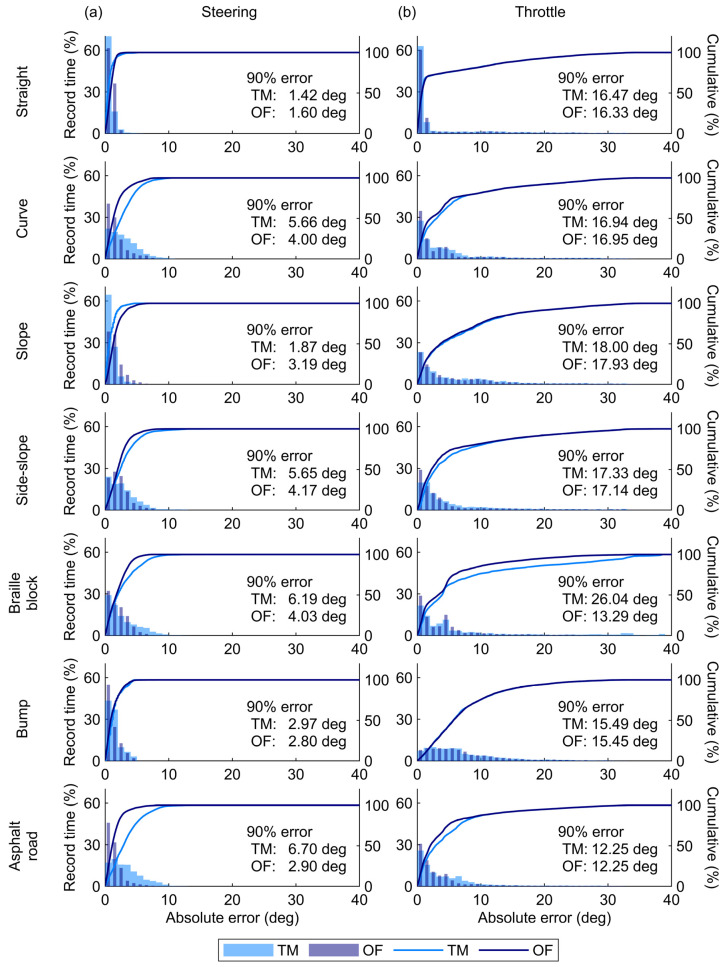
Histograms of the absolute error for (**a**) the steering angle and (**b**) the throttle angle. The dark blue and light blue bars/lines represent the estimated data obtained using the optical flow (OF) and template matching (TM) approaches, respectively. Each graph also indicates the 90th percentile error for each condition.

The time-series plots for the steering angle agreed with the true values (Figure 5a). The high accuracy of the steering angle estimation was also evident in the two-dimensional histograms of the true values and errors between the estimation and true values (Figure 6a). Across the entire range, the estimation errors were around zero degrees. Consequently, the 90th percentile errors for the steering angle were only several degrees (Figure 7a). These values were comparable to or better than those reported in a previous study [17] (Table 1), indicating that the monocular camera system successfully estimated the steering angle during MMS driving with practical accuracy.

The TM approach provided better accuracy of the steering angle under the straight and slope conditions (Figure 7a). The TM approach estimated the steering angle by calculating feature point shifts on a pixel-by-pixel basis through cross-correlation analysis of the luminance distribution between two images [23]. This process prevents variations in estimated values when the steering angle remains constant. In contrast, the OF approach computes feature point shifts with sub-pixel accuracy based on the optical flow constraint expressed as a continuous system [21]. This process enables the representation of smooth steering angle changes, resulting in high accuracy under the curve and side-slope conditions (Figure 7a).

**Figure 8:**
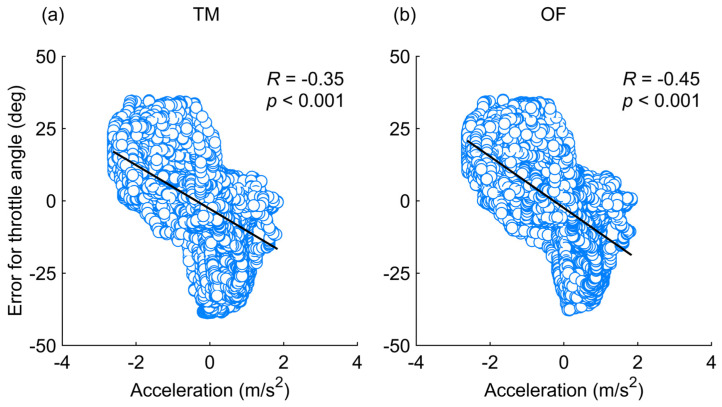
Correlation between the estimation errors (estimated value minus true value) for the throttle angle and forward acceleration using (**a**) template matching (TM) and (**b**) optical flow (OF) approaches. Each graph displays the Spearman’s rank correlation coefficient (*R*) and *p*-value.

The estimated throttle angle exhibited large errors during the phases with steep increases or decreases in the actual angles and under bump conditions (Figure 5b). The two-dimensional histograms in Figure 6b also illustrate that the estimation errors of the throttle angle varied depending on the operational status. The errors mostly remained close to zero degrees during the phase of maximum throttle angles but increased significantly when the throttle angle was near zero or partly at its maximum. Moreover, the correlation analysis in Figure 8 showed that high acceleration significantly increased the estimation errors for the throttle angle (*p* < 0.001). These results indicate that the difference between the vehicle’s actual speed and the set value—caused by vehicle inertia during acceleration and deceleration—led to large estimation errors in the throttle angle. Consequently, this difference significantly increased the 90th percentile errors of the throttle angle, sometimes exceeding tens of degrees (Figure 7b). In contrast, the estimation error of the throttle angle in the other phases was smaller than that at the start and end of MMS running. This smaller error suggests that the throttle angle was correctly estimated during periods with minimal discrepancy between vehicle motion and throttle operation.

## 4. Discussion

This study proposed a novel method for estimating the steering and throttle angles in MMS driving using a VO-based approach with monocular camera images. The VO-based method achieved better or comparable estimation accuracy for the steering angle compared with the previous IMU-based approach [17], as presented in Table 1. The IMU-based sensing system has been proposed as the primary method for monitoring user operations of MMSs and wheelchairs [15,16,17]. Unlike the VO-based method, IMU-based estimation is susceptible to sensor drift and acceleration noise caused by vehicle vibrations, negatively impacting estimation accuracy. Furthermore, the IMU-based method faces challenges related to sensor implementation including attachment fixation on the vehicle body and wiring between IMUs and data loggers, which can be problematic, particularly in settings where engineering staff are unavailable. In contrast, the VO-based system uses only a single monocular camera, unlike the IMU-based method that requires dual IMUs and a data logger, offering the advantage of a simpler measurement setup. Therefore, the proposed method enables accurate monitoring of the steering angle using a simple system, which offers a practical advantage for MMS driving evaluation in clinical settings where physical and occupational therapists with limited engineering knowledge use the measurement system.

Our study proposes the first VO-based approach that uses a single monocular recording to assess a user’s driving operations. In the field of vehicle driving recording systems, previous studies primarily used the Global Positioning System (GPS) [29]. Unlike GPS-based methods, the VO-based method enables application in indoor environments where GPS signals cannot be accurately recorded. The VO-based method also enables the estimation of vehicle operations through detailed measurements of vehicle movements, which are difficult to achieve using GPS alone. Furthermore, although previous studies developed VO-based methods for estimating vehicle movements from video images recorded by a monocular camera, they have focused primarily on autonomous driving technologies in the aerospace [30] and automotive fields [31]. Vision-based sensing systems using camera images have also been developed for MMSs and PWCs; however, they have primarily been applied to objective detection [18] or autonomous driving [32]. In contrast to these studies, our system serves as a fundamental VO-based technique for recording user operations in MMSs and PWCs that lack built-in logging systems.

This study confirmed that both the TM and OF approaches are applicable for estimating the steering angle in the MMS. In VO techniques, the TM and OF methods are commonly used for image processing [22]. However, the TM approach, which relies on relatively simple computations based on cross-correlation analysis of luminance distributions, is susceptible to noise, such as shadows in the image [24]. The OF approach, in contrast, may offer greater robustness against such noise and is better suited as a VO-based method for MMS recording. Further improvement in estimation accuracy may be achieved using advanced techniques in VO-based SLAM [33]. Sensor fusion approaches that integrate IMUs and/or wheel rotary encoders are well known to compensate for estimation errors arising from the limitations of individual sensors. However, unlike SLAM, which integrates velocity to estimate position and is therefore susceptible to error accumulation, the estimation of operation angles in this study relies directly on velocity values. This characteristic makes the calculation more tolerant of noise and errors in the velocity estimation via VO.

In future studies, the proposed technique is expected to contribute significantly to the collection of driving data for establishing quantitative evaluation measures of MMS operation. During such data collection, quantitative evidence regarding the reduced installation time of the VO-based system compared to the IMU-based system will be established. Its ease of implementation will be advantageous for large-scale data collection in real-life environments. Evidence-based evaluation of MMS driving skills may help address the lack of clinical knowledge of MMS usage highlighted by Mortensen and colleagues [10]. The previous studies of PWC have characterized joystick operation and evaluated driving skills and stability [16,34]. A similar quantitative approach is expected to be feasible for MMSs using the proposed system.

Several limitations of this study are noted. The VO technique cannot detect high-frequency throttle lever operations, such as sudden starts and stops of the MMS. Additional computational processing—such as compensating for the inertia effect based on further data collection—may be necessary to evaluate these detailed operations. Furthermore, the VO-based method remains challenging for practical use in clinical settings. First, the VO technique cannot eliminate the influence of driving vibrations and shadow reflections on estimation accuracy. In the proposed system, the image processing parameters of the OF approach were adjusted to prevent these adverse effects. However, these parameters may need to be optimized when applying the VO technique with different cameras or under varying conditions. Second, the proposed system requires camera calibration each time a monocular camera is attached to the MMS, which may confuse MMS users and the therapists supporting them. Practical usability could be improved by eliminating the camera calibration step and instead computing the calibration parameters directly from video images during MMS driving. Third, the experiment in the present study was conducted exclusively with young, healthy participants. This experimental design may have overlooked potential effects on estimation accuracy that could arise from the characteristic operation patterns of elderly individuals or persons with disabilities.

## 5. Conclusions

In this study, we proposed the VO-based method for logging the driving operation of MMSs, aiming for easier installation in clinical settings than operation logging with IMUs. The proposed method used a single monocular camera to successfully estimate the steering angle during MMS operations with comparable or superior accuracy to the IMU-based approach in a previous study, demonstrating its applicability for evaluating the driving skills and safety of MMS users. The estimation accuracy for the throttle angle was subject to the inertia effect of the MMS body but practical during stable driving. This method represents a fundamental technique for quantitative evaluation of user operations in MMSs and promotes safety use in clinical applications.

## Figures and Tables

**Table 1:** Comparison of the 90th percentile error in the estimation with those of a previous study.

Method	IMUs-Based Method [17]		VO-Based Method (This Study)			
			TM Approach		OF Approach	
Configuration	Dual IMUs and a Data Logger		Single Monocular Camera			
	Steering (deg)	Throttle (deg)	Steering (deg)	Throttle (deg)	Steering (deg)	Throttle (deg)
Straight	6.89	4.02	1.42	16.47	1.60	16.33
Curve	7.17	3.62	5.66	16.94	4.00	16.95
Slope	5.58	3.85	1.87	18.00	3.19	17.93
Side-slope	6.16	3.70	5.65	17.33	4.17	17.14
Braille block	8.16	4.95	6.19	26.04	4.03	13.29

## Data Availability

The data that support the findings of this study are available upon reasonable request to the corresponding author.
